# 6-Methyl-1-({[(2*E*)-2-methyl-3-phenyl­prop-2-en-1-yl]­oxy}meth­yl)-1,2,3,4-tetra­hydro­quinazoline-2,4-dione

**DOI:** 10.1107/S1600536812020429

**Published:** 2012-05-19

**Authors:** Nasser R. El-Brollosy, Mohamed I. Attia, Ali A. El-Emam, Seik Weng Ng, Edward R. T. Tiekink

**Affiliations:** a Department of Pharmaceutical Chemistry, College of Pharmacy, King Saud University, Riyadh 11451, Saudi Arabia; b Department of Chemistry, Faculty of Science, Tanta University, Tanta 31527, Egypt; c Department of Chemistry, University of Malaya, 50603 Kuala Lumpur, Malaysia; d Chemistry Department, Faculty of Science, King Abdulaziz University, PO Box 80203 Jeddah, Saudi Arabia

## Abstract

In the title compound, C_20_H_20_N_2_O_3_, the ten atoms comprising the quinazoline ring are essentially planar (r.m.s. deviation = 0.024 Å), and this plane is almost orthogonal to the terminal phenyl ring [dihedral angle = 82.87 (7)°]. The conformation about the ethyl­ene bond [1.335 (2) Å] is *E* and there is a significant twist between this residue and the adjacent phenyl ring [C—C—C— torsion angle = −48.4 (3)°]. The crystal structure features centrosymmetric dimeric units linked by pairs of N—H⋯O hydrogen bonds between the amide groups which lead to eight-membered {⋯HNCO}_2_ synthons. These are consolidated into a three-dimensional architecture by C—H⋯O, C—H⋯π and π–π inter­actions [centroid–centroid distances = 3.5087 (8) and 3.5645 (9) Å].

## Related literature

For background to non-nucleoside reverse transcriptase inhib­itors, see: Hopkins *et al.* (1996 ▶, 1999 ▶); El-Brollosy *et al.* (2008 ▶, 2009 ▶). For a related structure, see: El-Brollosy *et al.* (2012 ▶). For the synthesis, see: El-Brollosy (2007 ▶).
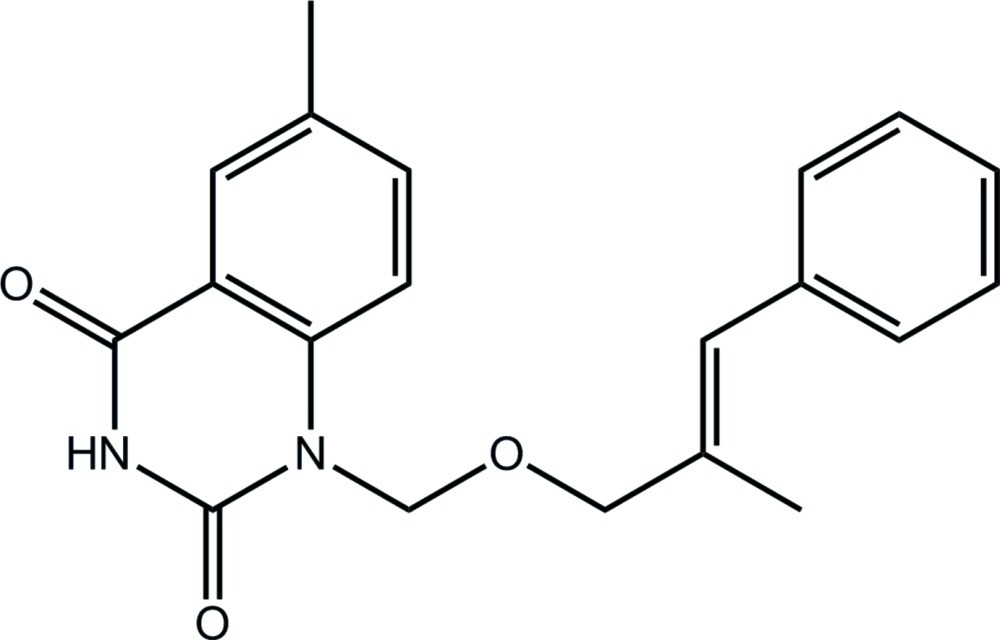


## Experimental

### 

#### Crystal data

C_20_H_20_N_2_O_3_
*M* *_r_* = 336.38
Monoclinic, 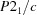

*a* = 16.2352 (8) Å
*b* = 13.6934 (6) Å
*c* = 7.8900 (4) Å
β = 102.606 (5)°
*V* = 1711.78 (14) Å^3^
*Z* = 4
Mo *K*α radiation
μ = 0.09 mm^−1^
*T* = 100 K
0.40 × 0.20 × 0.10 mm

#### Data collection

Agilent SuperNova Dual diffractometer with an Atlas detector
Absorption correction: multi-scan (*CrysAlis PRO*; Agilent, 2011 ▶) *T*_min_ = 0.522, *T*_max_ = 1.000
13993 measured reflections
3965 independent reflections
3067 reflections with *I* > 2σ(*I*)
*R*_int_ = 0.048

#### Refinement

*R*[*F* ^2^ > 2σ(*F* ^2^)] = 0.046
*wR*(*F* ^2^) = 0.129
*S* = 1.02
3965 reflections
232 parameters
H atoms treated by a mixture of independent and constrained refinement
Δρ_max_ = 0.28 e Å^−3^
Δρ_min_ = −0.25 e Å^−3^

### 

Data collection: *CrysAlis PRO* (Agilent, 2011 ▶); cell refinement: *CrysAlis PRO*; data reduction: *CrysAlis PRO*; program(s) used to solve structure: *SHELXS97* (Sheldrick, 2008 ▶); program(s) used to refine structure: *SHELXL97* (Sheldrick, 2008 ▶); molecular graphics: *ORTEP-3* (Farrugia, 1997 ▶) and *DIAMOND* (Brandenburg, 2006 ▶); software used to prepare material for publication: *publCIF* (Westrip, 2010 ▶).

## Supplementary Material

Crystal structure: contains datablock(s) global, I. DOI: 10.1107/S1600536812020429/hg5224sup1.cif

Structure factors: contains datablock(s) I. DOI: 10.1107/S1600536812020429/hg5224Isup2.hkl

Supplementary material file. DOI: 10.1107/S1600536812020429/hg5224Isup3.cml

Additional supplementary materials:  crystallographic information; 3D view; checkCIF report

## Figures and Tables

**Table 1:** Hydrogen-bond geometry (Å, °) *Cg*2 and *Cg*3 are the centroids of the C8–C8 and C15–C20 benzene rings, respectively.

*D*—H⋯*A*	*D*—H	H⋯*A*	*D*⋯*A*	*D*—H⋯*A*
N1—H1*n*⋯O2^i^	0.93 (2)	1.89 (2)	2.8180 (16)	172.9 (17)
C10—H10*B*⋯O1^ii^	0.99	2.49	3.3001 (18)	139
C11—H11*B*⋯O3^iii^	0.99	2.56	3.4462 (18)	150
C14—H14⋯*Cg*3^iv^	0.95	2.85	3.5574 (18)	132
C18—H18⋯*Cg*2^iv^	0.95	2.91	3.680 (2)	139

Symmetry codes: (i) 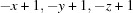; (ii) 
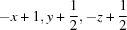
; (iii) 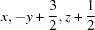; (iv) 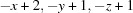.

**Fig. 1.**
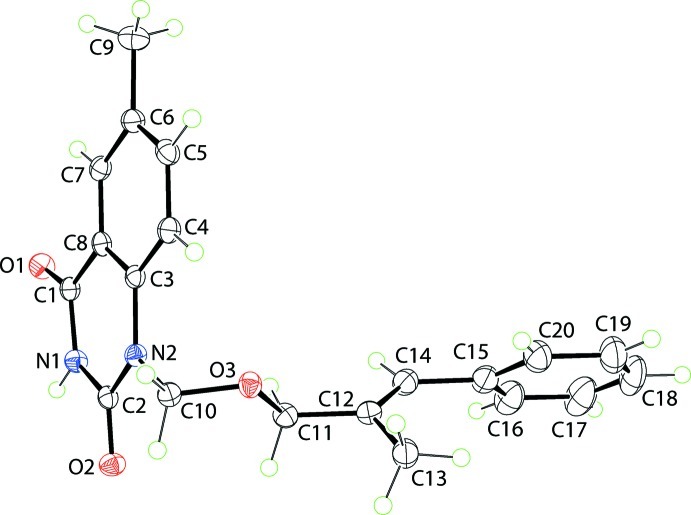
The molecular structure of (I) showing the atom-labelling scheme and displacement ellipsoids at the 50% probability level.

**Fig. 2.**
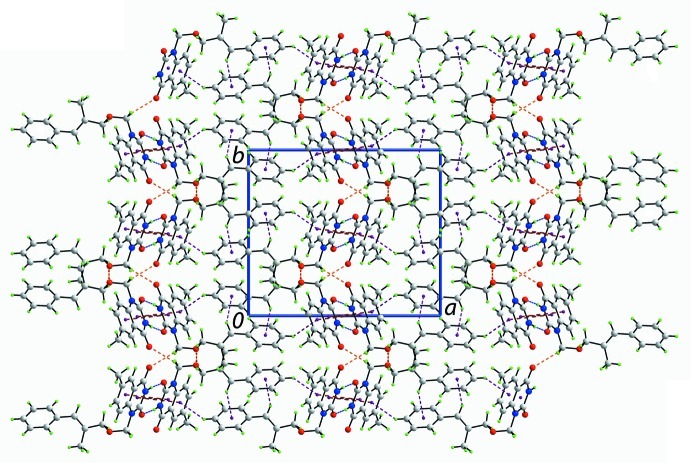
A view in projection down the c axis of the unit-cell contents for (I). The N—H···O, C—H···O, C—H···π and π—π interactions are shown as blue, orange, purple and brown dashed lines, respectively.

## supplementary crystallographic information

### Comment

In continuation of our interest in chemistry of non-nucleoside reverse transcriptase inhibitors (NNRTI's) (El-Brollosy *et al.*, 2008; El-Brollosy *et al.*, 2009), relevant to the treatment of human immunodeficiency virus (HIV) (Hopkins *et al.*, 1996; Hopkins *et al.*, 1999), we synthesized the title compound, 6-methyl-1-[((*E*)-2-methyl-3-phenylallyloxy)methyl]quinazoline-2,4(1*H*,3*H*)-dione (I), as a potential NNRTI (El-Brollosy, 2007). Herein, we describe the results of its crystal structure determination to complement the structure determination of the recently determined chloro analogue (El-Brollosy *et al.*, 2012).

The 10 atoms comprising the quinazoline ring in (I), Fig. 1, are co-planar with a r.m.s. = 0.024 Å; the maximum deviations from their least-squares plane are 0.036 (1) Å for the C2 atom and -0.032 (1) Å for the N2 atom. The dihedral angle between the fused ring system and the terminal phenyl ring of 82.87 (7)° is consistent with an almost orthogonal relationship. The conformation about the ethylene bond [C12═C14 = 1.335 (2) Å] is *E*. The torsion angle between the ethylene and phenyl rings, *i.e*. C12—C14—C15—C16, of -48.4 (3)° indicates a significant twist about the C14—C15 bond. Overall, the molecule in (I) is significantly more twisted than that observed in the chloro analogue (El-Brollosy *et al.*, 2012).

In the crystal structure, centrosymmetrically related molecules are connected *via* N—H···O hydrogen bonds between the amide groups (involving the carbonyl-O closest to the tertiary-N atom) which lead to eight-membered {···HNCO}_2_ synthons, Table 1. The dimeric aggregates are consolidated into a three-dimensional architecture by C—H···O and C—H···π interactions, Table 1, as well as by π—π contacts [ring centroid(N1,N2,C1–C3,C8)···centroid(N1,N2,C1–C3,C8)^i^ = 3.5087 (8) Å and tilt angle = 0° and ring centroid(N1,N2,C1–C3,C8)···centroid(C3–C8)^i^ = 3.5645 (9) Å and tilt angle = 1.85 (7)°, for symmetry operation *i*: 1 - *x*, 1 - *y*, -*z*). Globally, the crystal structure comprises alternating layers of quinazoline rings and 2-methyl-3-phenylallyloxy)methyl residues that stack along the *a* axis, Fig. 2.

### Experimental

6-Methylquinazoline-2,4(1*H*,3*H*)dione (0.176 g, 1 mmol) was stirred in dry acetonitrile (15 ml) under nitrogen and *N*,*O*-bis(trimethylsilyl)acetamide (0.87 ml, 3.5 mmol) was added. After a clear solution was obtained (10 min), the mixture was cooled to 223 K and trimethylsilyl trifluoromethanesulfonate (0.18 ml, 1 mmol) was added followed by the drop-wise addition of bis[(*E*)-2-methyl-3-phenylallyloxy]methane (0.616 g, 2 mmol). The reaction mixture was stirred at room temperature for 5 h, after which the reaction was quenched by the addition of sat. aq. NaHCO_3_ solution (5 ml). The mixture was evaporated under reduced pressure and the residue was extracted with ether (3 × 50 ml). The combined ether fractions were collected, dried (MgSO_4_) and evaporated under reduced pressure. The product was purified on silica gel column chromatography, using 20% ether in petroleum ether (40–60°C), to afford the title compound as a white solid in 78% yield (0.262 g). Single crystals were achieved by recrystallization from its ethanol solution (El-Brollosy 2007).

### Refinement

Carbon-bound H-atoms were placed in calculated positions [C—H = 0.95 to 0.99 Å, *U*_iso_(H) = 1.2–1.5*U*_eq_(C)] and were included in the refinement in the riding model approximation. The amino H-atom was refined freely.

### Crystal data

**Table:**

C_20_H_20_N_2_O_3_	*F*(000) = 712
*M**_r_* = 336.38	*D*_x_ = 1.305 Mg m^−^^3^
Monoclinic, *P*2_1_/*c*	Mo *K*α radiation, λ = 0.71073 Å
Hall symbol: -P 2ybc	Cell parameters from 4710 reflections
*a* = 16.2352 (8) Å	θ = 2.6–27.5°
*b* = 13.6934 (6) Å	µ = 0.09 mm^−^^1^
*c* = 7.8900 (4) Å	*T* = 100 K
β = 102.606 (5)°	Prism, colourless
*V* = 1711.78 (14) Å^3^	0.40 × 0.20 × 0.10 mm
*Z* = 4	

### Data collection

**Table:**

Agilent SuperNova Dual diffractometer with an Atlas detector	3965 independent reflections
Radiation source: SuperNova (Mo) X-ray Source	3067 reflections with *I* > 2σ(*I*)
Mirror monochromator	*R*_int_ = 0.048
Detector resolution: 10.4041 pixels mm^-1^	θ_max_ = 27.6°, θ_min_ = 2.6°
ω scan	*h* = −21→20
Absorption correction: multi-scan (*CrysAlis PRO*; Agilent, 2011)	*k* = −17→17
*T*_min_ = 0.522, *T*_max_ = 1.000	*l* = −10→8
13993 measured reflections	

### Refinement

**Table:**

Refinement on *F*^2^	Primary atom site location: structure-invariant direct methods
Least-squares matrix: full	Secondary atom site location: difference Fourier map
*R*[*F*^2^ > 2σ(*F*^2^)] = 0.046	Hydrogen site location: inferred from neighbouring sites
*wR*(*F*^2^) = 0.129	H atoms treated by a mixture of independent and constrained refinement
*S* = 1.02	*w* = 1/[σ^2^(*F*_o_^2^) + (0.0571*P*)^2^ + 0.6389*P*] where *P* = (*F*_o_^2^ + 2*F*_c_^2^)/3
3965 reflections	(Δ/σ)_max_ < 0.001
232 parameters	Δρ_max_ = 0.28 e Å^−^^3^
0 restraints	Δρ_min_ = −0.25 e Å^−^^3^

### Special details

**Table:**

Geometry. All e.s.d.'s (except the e.s.d. in the dihedral angle between two l.s. planes) are estimated using the full covariance matrix. The cell e.s.d.'s are taken into account individually in the estimation of e.s.d.'s in distances, angles and torsion angles; correlations between e.s.d.'s in cell parameters are only used when they are defined by crystal symmetry. An approximate (isotropic) treatment of cell e.s.d.'s is used for estimating e.s.d.'s involving l.s. planes.
Refinement. Refinement of *F*^2^ against ALL reflections. The weighted *R*-factor *wR* and goodness of fit *S* are based on *F*^2^, conventional *R*-factors *R* are based on *F*, with *F* set to zero for negative *F*^2^. The threshold expression of *F*^2^ > σ(*F*^2^) is used only for calculating *R*-factors(gt) *etc*. and is not relevant to the choice of reflections for refinement. *R*-factors based on *F*^2^ are statistically about twice as large as those based on *F*, and *R*- factors based on ALL data will be even larger.

### Fractional atomic coordinates and isotropic or equivalent isotropic displacement parameters (Å^2^)

**Table:**

	*x*	*y*	*z*	*U*_iso_*/*U*_eq_	
O1	0.51870 (7)	0.31276 (7)	0.15509 (14)	0.0230 (3)	
O2	0.55774 (7)	0.59676 (8)	0.45728 (13)	0.0209 (2)	
O3	0.72545 (6)	0.70049 (7)	0.27942 (13)	0.0190 (2)	
N1	0.53851 (8)	0.45651 (9)	0.30019 (16)	0.0180 (3)	
H1n	0.5078 (12)	0.4335 (14)	0.379 (2)	0.030 (5)*	
N2	0.60917 (7)	0.58944 (9)	0.21014 (16)	0.0162 (3)	
C1	0.54810 (9)	0.39555 (10)	0.16689 (19)	0.0174 (3)	
C2	0.56805 (9)	0.55065 (10)	0.32956 (18)	0.0169 (3)	
C3	0.62603 (9)	0.53339 (11)	0.07198 (18)	0.0163 (3)	
C4	0.67248 (9)	0.57169 (11)	−0.04270 (19)	0.0186 (3)	
H4	0.6932	0.6367	−0.0284	0.022*	
C5	0.68806 (9)	0.51480 (11)	−0.17644 (19)	0.0204 (3)	
H5	0.7198	0.5418	−0.2530	0.024*	
C6	0.65885 (9)	0.41867 (11)	−0.20352 (19)	0.0202 (3)	
C7	0.61195 (9)	0.38169 (11)	−0.09098 (19)	0.0189 (3)	
H7	0.5901	0.3172	−0.1078	0.023*	
C8	0.59609 (9)	0.43757 (11)	0.04694 (18)	0.0169 (3)	
C9	0.67858 (11)	0.35752 (12)	−0.3488 (2)	0.0270 (4)	
H9A	0.6299	0.3162	−0.3977	0.041*	
H9B	0.7277	0.3162	−0.3034	0.041*	
H9C	0.6909	0.4004	−0.4396	0.041*	
C10	0.63668 (9)	0.69150 (10)	0.23222 (19)	0.0176 (3)	
H10A	0.6155	0.7272	0.1222	0.021*	
H10B	0.6117	0.7221	0.3230	0.021*	
C11	0.76134 (9)	0.65578 (11)	0.44431 (19)	0.0201 (3)	
H11A	0.7536	0.5841	0.4354	0.024*	
H11B	0.7321	0.6802	0.5336	0.024*	
C12	0.85380 (10)	0.67948 (11)	0.49682 (19)	0.0213 (3)	
C13	0.87568 (10)	0.78634 (12)	0.5069 (2)	0.0258 (4)	
H13A	0.9351	0.7945	0.5650	0.039*	
H13B	0.8399	0.8206	0.5729	0.039*	
H13C	0.8663	0.8135	0.3893	0.039*	
C14	0.90837 (10)	0.60631 (12)	0.5433 (2)	0.0248 (4)	
H14	0.8855	0.5422	0.5298	0.030*	
C15	1.00037 (10)	0.61367 (12)	0.6133 (2)	0.0289 (4)	
C16	1.03668 (12)	0.55823 (14)	0.7593 (3)	0.0376 (4)	
H16	1.0020	0.5168	0.8105	0.045*	
C17	1.12270 (13)	0.56292 (15)	0.8303 (3)	0.0468 (5)	
H17	1.1464	0.5257	0.9307	0.056*	
C18	1.17373 (12)	0.62174 (16)	0.7550 (3)	0.0481 (6)	
H18	1.2326	0.6249	0.8038	0.058*	
C19	1.13958 (12)	0.67605 (15)	0.6089 (3)	0.0430 (5)	
H19	1.1749	0.7163	0.5570	0.052*	
C20	1.05311 (11)	0.67162 (14)	0.5379 (3)	0.0342 (4)	
H20	1.0299	0.7086	0.4368	0.041*	

### Atomic displacement parameters (Å^2^)

**Table:**

	*U*^11^	*U*^22^	*U*^33^	*U*^12^	*U*^13^	*U*^23^
O1	0.0252 (6)	0.0164 (5)	0.0286 (6)	−0.0030 (4)	0.0084 (5)	0.0003 (4)
O2	0.0243 (6)	0.0206 (5)	0.0193 (6)	−0.0032 (4)	0.0079 (4)	−0.0011 (4)
O3	0.0172 (5)	0.0208 (5)	0.0190 (5)	−0.0022 (4)	0.0041 (4)	0.0018 (4)
N1	0.0191 (6)	0.0181 (6)	0.0180 (6)	−0.0017 (5)	0.0064 (5)	0.0020 (5)
N2	0.0163 (6)	0.0156 (6)	0.0169 (6)	−0.0016 (5)	0.0039 (5)	−0.0002 (5)
C1	0.0152 (7)	0.0171 (7)	0.0190 (7)	0.0009 (6)	0.0021 (5)	0.0021 (6)
C2	0.0142 (7)	0.0183 (7)	0.0176 (7)	0.0013 (6)	0.0023 (5)	0.0012 (6)
C3	0.0138 (7)	0.0174 (7)	0.0165 (7)	0.0019 (6)	0.0005 (5)	0.0006 (5)
C4	0.0176 (7)	0.0176 (7)	0.0201 (7)	−0.0014 (6)	0.0029 (6)	0.0014 (6)
C5	0.0178 (7)	0.0246 (8)	0.0192 (7)	0.0016 (6)	0.0050 (6)	0.0035 (6)
C6	0.0191 (7)	0.0224 (8)	0.0185 (7)	0.0035 (6)	0.0031 (6)	−0.0002 (6)
C7	0.0179 (7)	0.0175 (7)	0.0195 (7)	0.0010 (6)	0.0003 (6)	0.0008 (6)
C8	0.0136 (7)	0.0171 (7)	0.0191 (7)	0.0017 (6)	0.0018 (5)	0.0027 (6)
C9	0.0333 (9)	0.0248 (8)	0.0247 (8)	0.0034 (7)	0.0103 (7)	−0.0021 (7)
C10	0.0179 (7)	0.0151 (7)	0.0198 (7)	−0.0005 (6)	0.0040 (5)	0.0000 (5)
C11	0.0205 (8)	0.0209 (8)	0.0191 (7)	0.0008 (6)	0.0050 (6)	0.0018 (6)
C12	0.0212 (8)	0.0249 (8)	0.0180 (7)	−0.0007 (6)	0.0046 (6)	−0.0024 (6)
C13	0.0207 (8)	0.0237 (8)	0.0321 (9)	−0.0002 (7)	0.0039 (6)	−0.0027 (7)
C14	0.0236 (8)	0.0240 (8)	0.0258 (8)	−0.0001 (7)	0.0031 (6)	−0.0028 (6)
C15	0.0240 (9)	0.0244 (8)	0.0356 (10)	0.0048 (7)	0.0008 (7)	−0.0077 (7)
C16	0.0331 (10)	0.0315 (10)	0.0426 (11)	0.0059 (8)	−0.0043 (8)	−0.0014 (8)
C17	0.0359 (11)	0.0375 (11)	0.0555 (13)	0.0104 (9)	−0.0148 (9)	−0.0033 (9)
C18	0.0217 (9)	0.0396 (11)	0.0736 (15)	0.0072 (9)	−0.0104 (9)	−0.0158 (11)
C19	0.0239 (9)	0.0392 (11)	0.0642 (14)	−0.0002 (8)	0.0057 (9)	−0.0135 (10)
C20	0.0250 (9)	0.0338 (10)	0.0419 (11)	0.0038 (8)	0.0034 (8)	−0.0059 (8)

### Geometric parameters (Å, º)

**Table:**

O1—C1	1.2257 (18)	C10—H10A	0.9900
O2—C2	1.2311 (17)	C10—H10B	0.9900
O3—C10	1.4130 (17)	C11—C12	1.503 (2)
O3—C11	1.4409 (18)	C11—H11A	0.9900
N1—C1	1.3780 (19)	C11—H11B	0.9900
N1—C2	1.3773 (19)	C12—C14	1.335 (2)
N1—H1n	0.93 (2)	C12—C13	1.504 (2)
N2—C2	1.3749 (18)	C13—H13A	0.9800
N2—C3	1.4081 (18)	C13—H13B	0.9800
N2—C10	1.4659 (18)	C13—H13C	0.9800
C1—C8	1.468 (2)	C14—C15	1.479 (2)
C3—C8	1.398 (2)	C14—H14	0.9500
C3—C4	1.400 (2)	C15—C20	1.393 (3)
C4—C5	1.379 (2)	C15—C16	1.397 (3)
C4—H4	0.9500	C16—C17	1.388 (3)
C5—C6	1.400 (2)	C16—H16	0.9500
C5—H5	0.9500	C17—C18	1.379 (3)
C6—C7	1.386 (2)	C17—H17	0.9500
C6—C9	1.509 (2)	C18—C19	1.382 (3)
C7—C8	1.399 (2)	C18—H18	0.9500
C7—H7	0.9500	C19—C20	1.395 (3)
C9—H9A	0.9800	C19—H19	0.9500
C9—H9B	0.9800	C20—H20	0.9500
C9—H9C	0.9800		
			
C10—O3—C11	112.91 (11)	O3—C10—H10B	109.1
C1—N1—C2	127.03 (13)	N2—C10—H10B	109.1
C1—N1—H1n	118.0 (12)	H10A—C10—H10B	107.9
C2—N1—H1n	114.9 (12)	O3—C11—C12	109.88 (12)
C2—N2—C3	121.70 (12)	O3—C11—H11A	109.7
C2—N2—C10	117.89 (12)	C12—C11—H11A	109.7
C3—N2—C10	120.40 (11)	O3—C11—H11B	109.7
O1—C1—N1	120.75 (13)	C12—C11—H11B	109.7
O1—C1—C8	124.49 (13)	H11A—C11—H11B	108.2
N1—C1—C8	114.76 (12)	C14—C12—C13	125.52 (14)
O2—C2—N2	122.40 (13)	C14—C12—C11	118.48 (14)
O2—C2—N1	120.88 (13)	C13—C12—C11	115.78 (13)
N2—C2—N1	116.72 (12)	C12—C13—H13A	109.5
C8—C3—C4	118.68 (13)	C12—C13—H13B	109.5
C8—C3—N2	120.03 (13)	H13A—C13—H13B	109.5
C4—C3—N2	121.29 (13)	C12—C13—H13C	109.5
C5—C4—C3	119.85 (13)	H13A—C13—H13C	109.5
C5—C4—H4	120.1	H13B—C13—H13C	109.5
C3—C4—H4	120.1	C12—C14—C15	127.40 (15)
C4—C5—C6	122.38 (14)	C12—C14—H14	116.3
C4—C5—H5	118.8	C15—C14—H14	116.3
C6—C5—H5	118.8	C20—C15—C16	118.26 (16)
C7—C6—C5	117.47 (14)	C20—C15—C14	122.97 (16)
C7—C6—C9	121.41 (14)	C16—C15—C14	118.75 (17)
C5—C6—C9	121.12 (14)	C17—C16—C15	120.9 (2)
C6—C7—C8	121.24 (14)	C17—C16—H16	119.5
C6—C7—H7	119.4	C15—C16—H16	119.5
C8—C7—H7	119.4	C18—C17—C16	119.9 (2)
C3—C8—C7	120.38 (13)	C18—C17—H17	120.0
C3—C8—C1	119.59 (13)	C16—C17—H17	120.0
C7—C8—C1	120.04 (13)	C17—C18—C19	120.27 (18)
C6—C9—H9A	109.5	C17—C18—H18	119.9
C6—C9—H9B	109.5	C19—C18—H18	119.9
H9A—C9—H9B	109.5	C18—C19—C20	119.8 (2)
C6—C9—H9C	109.5	C18—C19—H19	120.1
H9A—C9—H9C	109.5	C20—C19—H19	120.1
H9B—C9—H9C	109.5	C15—C20—C19	120.78 (18)
O3—C10—N2	112.40 (11)	C15—C20—H20	119.6
O3—C10—H10A	109.1	C19—C20—H20	119.6
N2—C10—H10A	109.1		
			
C2—N1—C1—O1	179.94 (14)	C6—C7—C8—C1	−178.37 (13)
C2—N1—C1—C8	0.7 (2)	O1—C1—C8—C3	179.38 (14)
C3—N2—C2—O2	175.15 (13)	N1—C1—C8—C3	−1.44 (19)
C10—N2—C2—O2	−3.8 (2)	O1—C1—C8—C7	−0.9 (2)
C3—N2—C2—N1	−4.7 (2)	N1—C1—C8—C7	178.28 (13)
C10—N2—C2—N1	176.31 (12)	C11—O3—C10—N2	−62.48 (15)
C1—N1—C2—O2	−177.56 (14)	C2—N2—C10—O3	111.14 (14)
C1—N1—C2—N2	2.3 (2)	C3—N2—C10—O3	−67.84 (16)
C2—N2—C3—C8	4.1 (2)	C10—O3—C11—C12	−172.58 (12)
C10—N2—C3—C8	−176.95 (12)	O3—C11—C12—C14	−128.83 (15)
C2—N2—C3—C4	−176.01 (13)	O3—C11—C12—C13	56.34 (17)
C10—N2—C3—C4	2.9 (2)	C13—C12—C14—C15	0.0 (3)
C8—C3—C4—C5	−0.3 (2)	C11—C12—C14—C15	−174.30 (15)
N2—C3—C4—C5	179.85 (13)	C12—C14—C15—C20	−48.4 (3)
C3—C4—C5—C6	0.1 (2)	C12—C14—C15—C16	133.26 (19)
C4—C5—C6—C7	0.7 (2)	C20—C15—C16—C17	1.9 (3)
C4—C5—C6—C9	−178.65 (14)	C14—C15—C16—C17	−179.73 (17)
C5—C6—C7—C8	−1.5 (2)	C15—C16—C17—C18	−1.0 (3)
C9—C6—C7—C8	177.92 (14)	C16—C17—C18—C19	0.0 (3)
C4—C3—C8—C7	−0.4 (2)	C17—C18—C19—C20	0.3 (3)
N2—C3—C8—C7	179.44 (13)	C16—C15—C20—C19	−1.6 (3)
C4—C3—C8—C1	179.28 (13)	C14—C15—C20—C19	−179.96 (16)
N2—C3—C8—C1	−0.8 (2)	C18—C19—C20—C15	0.6 (3)
C6—C7—C8—C3	1.3 (2)		

### Hydrogen-bond geometry (Å, º)

Cg2 and Cg3 are the centroids of the C8–C8 and C15–C20 benzene rings, respectively.

**Table:**

*D*—H···*A*	*D*—H	H···*A*	*D*···*A*	*D*—H···*A*
N1—H1*n*···O2^i^	0.93 (2)	1.89 (2)	2.8180 (16)	172.9 (17)
C10—H10*B*···O1^ii^	0.99	2.49	3.3001 (18)	139
C11—H11*B*···O3^iii^	0.99	2.56	3.4462 (18)	150
C14—H14···*Cg*3^iv^	0.95	2.85	3.5574 (18)	132
C18—H18···*Cg*2^iv^	0.95	2.91	3.680 (2)	139

Symmetry codes: (i) −*x*+1, −*y*+1, −*z*+1; (ii) −*x*+1, *y*+1/2, −*z*+1/2; (iii) *x*, −*y*+3/2, *z*+1/2; (iv) −*x*+2, −*y*+1, −*z*+1.
